# Advances in gene therapy for inborn errors of immunity

**DOI:** 10.1097/ACI.0000000000000952

**Published:** 2023-10-13

**Authors:** Lisa M. Ott de Bruin, Arjan C. Lankester, Frank J.T. Staal

**Affiliations:** a Willem-Alexander Children's Hospital, Department of Pediatrics, Pediatric Stem Cell Transplantation Program and Laboratory for Pediatric Immunology; b Department of Immunology, Leiden University Medical Center, Leiden, The Netherlands

**Keywords:** gene editing, gene therapy, IEI, inborn errors of immunity, PID, primary immunodeficiency, viral gene addition

## Abstract

**Purpose of review:**

Provide an overview of the landmark accomplishments and state of the art of gene therapy for inborn errors of immunity (IEI).

**Recent findings:**

Three decades after the first clinical application of gene therapy for IEI, there is one market authorized product available, while for several others efficacy has been demonstrated or is currently being tested in ongoing clinical trials. Gene editing approaches using programmable nucleases are being explored preclinically and could be beneficial for genes requiring tightly regulated expression, gain-of-function mutations and dominant-negative mutations.

**Summary:**

Gene therapy by modifying autologous hematopoietic stem cells (HSCs) offers an attractive alternative to allogeneic hematopoietic stem cell transplantation (HSCT), the current standard of care to treat severe IEI. This approach does not require availability of a suitable allogeneic donor and eliminates the risk of graft versus host disease (GvHD). Gene therapy can be attempted by using a viral vector to add a copy of the therapeutic gene (viral gene addition) or by using programmable nucleases (gene editing) to precisely correct mutations, disrupt a gene or introduce an entire copy of a gene at a specific locus. However, gene therapy comes with its own challenges such as safety, therapeutic effectiveness and access. For viral gene addition, a major safety concern is vector-related insertional mutagenesis, although this has been greatly reduced with the introduction of safer vectors. For gene editing, the risk of off-site mutagenesis is a main driver behind the ongoing search for modified nucleases. For both approaches, HSCs have to be manipulated *ex vivo,* and doing this efficiently without losing stemness remains a challenge, especially for gene editing.

## INTRODUCTION

Inborn errors of immunity (IEI) are a heterogeneous group of monogenetic disorders that affect the innate or adaptive immune system. Clinical manifestations may include severe, atypical or recurrent infections as well as immune dysregulation characterized by autoimmunity, autoinflammation, atopy, and increased risk of cancer. To date, approximately 500 IEI causing genes have been described, and with the widely available sequencing methods this number continues to grow rapidly [1]. Established treatment options consist of immune modulatory drugs, antimicrobial prophylaxis, immunoglobulin replacement therapy and, for severe IEI, allogeneic hematopoietic stem cell transplantation (HSCT). With HSCT, a patient's hematopoietic stem cells (HSCs) are replaced by healthy donor HSCs. The first successful HSCT were performed in 1968, to treat severe combined immunodeficiency (SCID) [2,3]. HSCT emerged as a life-saving therapy, but often resulted in partial or insufficient cure, with high morbidity and mortality, particularly in the large subgroup of patients dependent on treatment with a mismatched or haploidentical donor. Unfavorable HSCT outcomes with mismatched donors, growing knowledge on the genetic causes of SCID and the recognition of revertants as naturally occurring phenomenon leading to correction of the disease-causing genetic defect [4,5] together prompted the development of gene therapy in the 1990s. Gene therapy aims to correct a patient's own HSCs, thereby avoiding any risk of alloreactivity related complications [6]. X-linked SCID (X-SCID) and adenosine deaminase-deficient severe combined immunodeficiency (ADA-SCID) were the first genetic diseases to be successfully treated by gene therapy [7–13] eventually resulting in Strimvelis as the first, and to date only, *ex vivo* gene therapy with market authorization for the treatment of ADA-SCID [14]. In the past decades new viral vectors with a more favorable safety profile have been developed, integration site analysis techniques for safety monitoring have improved and conditioning is increasingly being used to facilitate engraftment of the corrected cells. Still, several challenges remain, most importantly the efficacy, safety and access to gene therapy.

In parallel with the development of gene therapy, the field of HSCT has made great strides since the 1990s, with improved human leukocyte antigen (HLA) typing and donor selection strategies, more effective and less toxic conditioning regimens and innovation in stem cell graft manipulation. Advances were also made in the management of toxicities, graft versus host disease (GvHD) and infections. However, HSCT for IEI still comes with a significant risk of morbidity and mortality in up to 25% of the patients, particularly in those patients transplanted with a mismatched donor [15–21].

This review will summarize the main aspects of gene therapy in IEI, the progress made and the remaining challenges. 

**Box 1:**
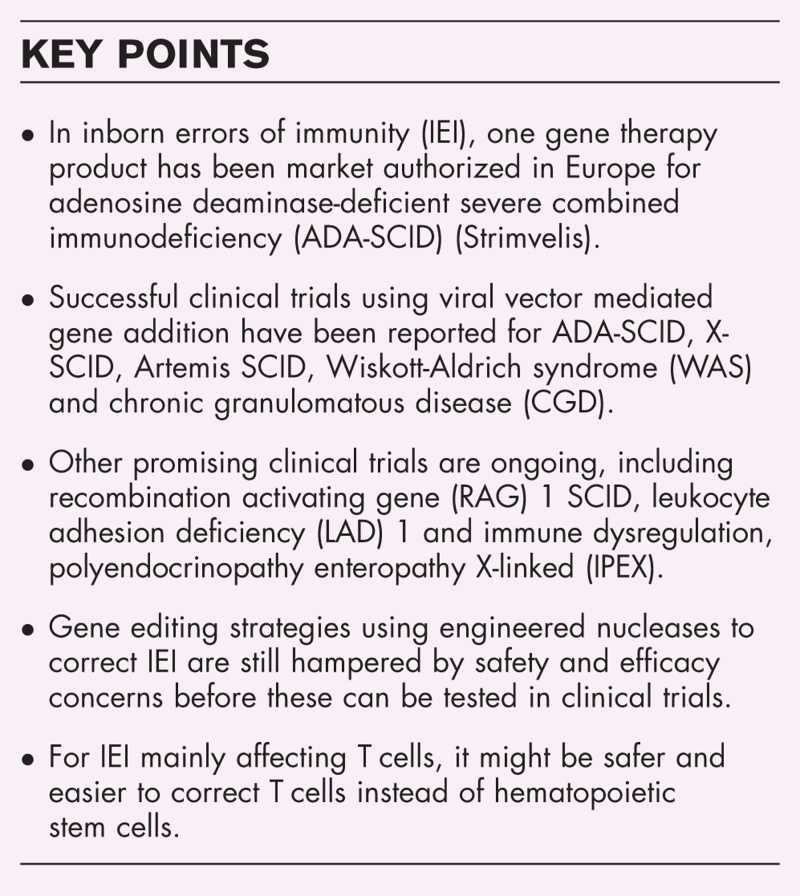
no caption available

## GENE THERAPY

**FIGURE 1:**
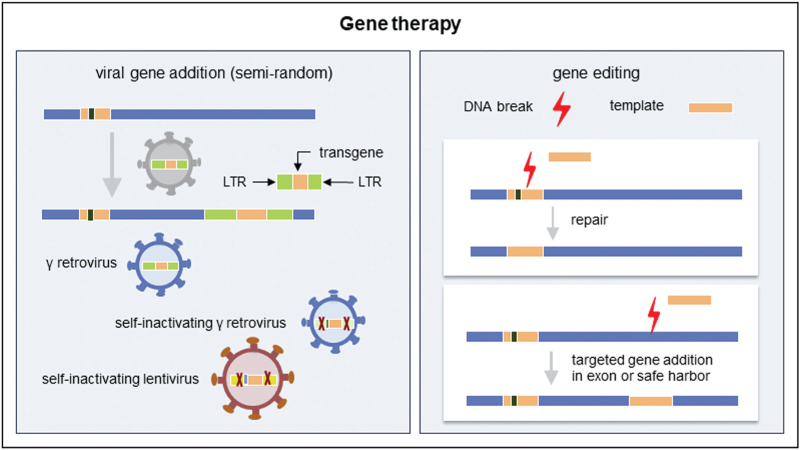
Different approaches to gene therapy for IEI. In all types of gene therapy for IEI, *ex vivo* gene therapy is used, in which a viral vector is used to genetically modify cells from the patients with therapeutic intend. Cells could include T cells, HSCs or other cell types. For gene addition (left), gRV or LV vectors are used to add the therapeutic gene to the genome of the target cells. This occurs semi-randomly, with slight preferences for integration sites depending on the vector used. For gene editing, a specific site can be targeted to change the DNA and correct the mutation. This can be accomplished by using an engineered nuclease such as CRISPR/Cas to introduce a single or double stranded DNA break in combination with a repair template. This strategy can be used to add a full cDNA in the same gene under control of the endogenous regulatory elements. Alternatively, it can be used to add a full cDNA including regulatory elements into a known safe region of the genome elsewhere (safe harbor). This targeted approach avoids insertional mutagenesis, but allows gene expression to be controlled by design. gRV, gamma-retroviruses; HSC, hematopoietic stem cell; IEI, inborn errors of immunity; LV, lentiviral.

Currently, all clinically tested gene therapy strategies for IEI rely on gene addition using viral gene transfer vectors (Table 1). Newer methods, based on gene editing with engineered nucleases, are still at a preclinical stage. In the next section, the two different approaches will be explained, with a focus on viral gene addition (Fig. 1). Importantly, regardless of the strategy, the autologous HSCs need to be collected, purified and manipulated *ex vivo* in optimal culture conditions to maintain their stemness characteristics [22]. Just as for HSCT, autologous HSCs can be obtained from peripheral blood by leukapheresis or from bone marrow by aspiration. Prior to leukapheresis patients are generally treated with a combination of GCS-F and plerixafor (CXCR4 antagonist) to mobilize their HSCs from bone marrow to peripheral blood [23]. The mobilized HSC approach is increasingly applied as this results in a much higher HSC yield which has a positive impact on hematological and immunological recovery [24]. Although initial SCID gene therapy studies were performed without conditioning it has become clear, similar to what is known for regular HSCT, that a certain level of myeloablative conditioning is required for successful and sustainable engraftment of corrected cells [8,25–28]. The amount and type of conditioning needed depends on the underlying disease and the selective advantage of the corrected cells [29].

**Table 1:** Gene therapies using viral gene addition

Disease name	Gene	Vector	Status	Ref/trial
ADA-SCID	ADA	gRV	Commercial product in Europe (strimvelis)	[14]
	ADA	LV	Compassionate use (GOSH)/Human trial (UCLA/NIH)	[79^▪▪^]
X-SCID	IL2RG	SIN gRV	Human trial completed	[37]
	IL2RG	LV	Human trial GOSH/BCH/UCL open	[82,83] NCT03311503, NCT03601286
	IL2RG	LV	Human trial UCSF/St Jude/Seattle/NIH Suspended	NCT01306019 NCT01512888
Artemis SCID	DCLRE1C	LV	Human trial UCSF Open	[86^▪▪^] NCT03538899
	DCLRE1C	LV	Human trial Paris Necker hospital Open	NCT05071222
RAG1 SCID	RAG1	LV	Human trial Open LUMC	NCT04797260 [125]
RAG2 SCID	RAG2	LV	Mice	[126,127] (manuscript in preparation)
WAS	WAS	LV	Human trial completed	[90,91,92^▪^]
X-CGD	CYBB	LV	Human trial (UCLA and UCL/GOSH) Open	[96] NCT01855685 NCT02234934
Autosomal recessive CGD	NCF1	LV	Human trial at GOSH, starting soon at NIH	NCT05207657
LAD1	ITGB2	LV	Human trial (UCLA, HIUNJ, GOSH) Open	NCT03812263 [106]
IPEX	FOXP3	LV in T cells	Ongoing Human trial (Stanford)	NCT05241444 [110,111]
fHLH	PRF1	gRV in T cells	Mice	[128]
	PRF1	LV	Mice	[129]
	UNC13D	LV	Mice (Stanford)	[130–133]
XIAP	XIAP	LV	Mice	[134]
XLP1	SAP	LV	Mice	[135]
	SAP	gRV in T cells	Mice	[136]
XLA	BTK	LV	Mice	[137,138]
Reticular dysgenesis	AK2	LV	In vitro	[139]

ADA-SCID, adenosine deaminase-deficient severe combined immunodeficiency (SCID); CGD, chronic granulomatous disease; fHLH, familial hemophagocytic lymphohistiocytosis; gRV, gamma-retroviral; IPEX, immune dysregulation, polyendocrinopathy enteropathy X-linked; LAD, leukocyte adhesion deficiency; LV, lentiviral; RAG, recombination activating gene; WAS, Wiskott-Aldrich syndrome; XIAP, X-linked inhibitor of apoptosis protein deficiency; XLA, X-linked agammaglobulinemia; XLP, X-linked lymphoproliferative disease; X-SCID, X-linked SCID.

### Viral gene addition

Virus based vectors make use of the ability of RNA viruses to infect and stably integrate genetic material into the target cell's genome. These vectors contain a correct copy of the therapeutic gene (transgene) along with regulatory elements that control gene expression, such as promoters and enhancers (Fig. 1). The genes necessary for viral replication have been removed from these vectors, while maintaining features required for gene transfer, such as the LTR (long terminal repeat). This way, viral vectors can infect and stably transfer a therapeutic transgene into the patient HSC's genome (transduction). In general, the insertion of the transgene happens semi-randomly throughout the host's genome, but different viruses have different preferences thereby giving rise to a different integration pattern [30]. The number of viral vector transgenes integrated in the genome per cell is referred to as vector copy number (VCN) [31]. This is usually determined at population level and not at a single cell basis. The required therapeutic protein level depends on the disease and is determined by both VCN and strength of the regulatory elements of the transgene.

The first clinical trials of gene therapy used gamma-retroviruses (gRV) and successful T cell reconstitution was achieved for X-SCID [7,12], ADA [8,11] and WAS [32,33]. Unfortunately, several patients developed hematological malignancies due to preferential integration near transcription initiation sites of other genes (including oncogenes). The strong enhancer activity of the viral LTR in a number of cases led to increased and deregulated expression of the nearby oncogenes [33–36]. Newer generations of self-inactivating (SIN) gRV vectors have been developed with deletion of enhancer elements of the LTR, which resulted in better safety [37]. In recent years, clinical trials have predominantly used SIN-lentiviral (LV) vectors [38], with LTRs without enhancer and weaker or lineage-specific promoters. In addition, the insertional pattern of LV vectors seems safer than that of gRV vectors, because LV have no insertional preference for transcription initiation sites [30]. Lastly, LV transduction also works on quiescent HSCs enabling retention of stemness [38].

Safety assays to test for genotoxicity of viral vectors are paramount in both the preclinical and clinical studies, and several are currently being used [39]. In the most commonly used In Vitro Immortalization (IVIM) assay, HSCs are expanded after transduction with retroviral vectors. Following limiting dilution, nonimmortalized cells stop proliferating, whereas insertional mutants give rise to clonal outgrowth. With the surrogate assay for genotoxicity assessment (SAGA), the gene expression profile of transduced murine HSCs is analyzed and machine learning is used to detect dysregulated genes as immortalization signature. In addition, advanced integration site analysis using next generation sequencing enables researchers and clinicians to monitor patients enrolled in clinical trials closely and detect any preferential vector integration pattern leading to clonal outgrowth at a very early stage. To date, no hematological malignancies have been reported in patients with IEI treated with LV vector gene therapy, but the risk still remains that insertional mutagenic events occur.

### Gene editing

Over the last decade the field of gene editing has grown exponentially [40]. This technique introduces a single (nick) or double strand DNA break (DSB) at a specific target site in the DNA and relies on the natural process of homology directed repair to introduce a template sequence (donor template or repair template). These nicks or breaks are made using engineered nucleases such as zinc-finger nuclease (ZFN), transcription activator-like effector nucleases (TALEN) and CRISPR-Cas. Gene editing can be used to correct a specific mutation at the endogenous locus, which has been done *in vitro* for X-CGD [41,42] and RAG2 [43,44] or to add the therapeutic gene to the same locus under the control of its endogenous promoter. Proof of principle of this approach has been demonstrated for X-SCID [45–47], X-CGD [48], CD40L [49^▪^,^50^,^51^], CTLA4 [52], XLP [53], RAG1 [54,55] and Wiskott–Aldrich syndrome (WAS) [56]. With both approaches, endogenous gene regulation is kept intact resulting in physiological expression. This is crucial when transcriptional control of the gene is tightly regulated, as in the case of CD40L, where constitutive expression leads to lymphoproliferative disorders [57,58].

Alternatively, a correct copy plus gene regulation elements can be added to a well studied safe harbor locus elsewhere in the genome [59]. In contrast to viral gene addition, with insertion happening semi-randomly, the location of insertion can now be specifically chosen, eliminating the risk of insertional mutagenesis (Fig. 1).

In the absence of a homologous donor template, a cell can repair the DSB by nonhomologous end joining (NHEJ). NHEJ is error-prone and may therefore lead to allele inactivation, thus potentially correcting gain-of-function and dominant-negative mutations or inducing a therapeutic knockout [60]. NHEJ occurs at a much higher rate than homology directed repair. Therefore, allele inactivation is much easier to accomplish than correction with gene editing using engineered nucleases [61].

Even though engineered nucleases are designed to target a specific site, there's still the risk of introducing unwanted genomic alterations (on or off-site mutagenesis) leading to genotoxicity. Over the last decade new nuclease variants have been generated to reduce off- and on-target adverse effects [62,63] and tools to evaluate these risks [64]. Currently, homology directed repair is still inefficient and generally less efficient than viral gene addition, making it only suitable for diseases with a high selective advantage for corrected cells. In addition, delivering the (modified) nuclease and the donor template into the HSCs efficiently without losing the HSCs pluripotent potential is still challenging, especially when aiming to introduce an entire copy of a therapeutic gene and not just a short fragment to correct a specific region only. The last decade, several methods have been optimized to introduce the programmed nucleases efficiently to the cells [65], but the large size of the donor template makes most nonviral methods inadequate for the donor template. The most commonly used successful strategy has been delivery by AAV6 [45,47,48,49^▪^,^51^–^53^], an adeno-associated nonintegrating viral vector. The safety of this approach and the potential risk for AAV integration needs to be further evaluated before clinical translation [66^▪^].

Prime and base editing strategies are being developed that use impaired Cas or TALEN variants that edit the mutation directly, without introducing a DSB or nick and without the need for any donor template [67,68]. As a proof of concept, base editors have been used in patient cells to correct one of the most common heterozygous STAT3 mutations causing hyper IgE syndrome [69]. Although prime and base editors are appealing from a safety perspective, they require a different strategy for each mutation, making them less suitable as therapeutic option for most IEI as these are all rare diseases caused by many different mutations.

## GENE THERAPY FOR SEVERE COMBINED IMMUNODEFICIENCY

Severe combined immunodeficiencies (SCIDs) can be caused by different genetic defects resulting in defective differentiation of HSCs into T cells. Natural killer (NK) cells and B cells can be absent as well and although B cells can be present, those are always dysfunctional due to either intrinsic defects or lack of proper T cell help. These patients lack adaptive immunity completely and require curative treatment as early as possible or they will succumb to life-threatening infections within the first year of life. The implementation of TREC newborn screening in many countries has resulted in earlier detection and treatment [70].

Gene therapy is currently tested in clinical trials for ADA-SCID, X-linked SCID, Artemis SCID and RAG1 SCID. In Europe it is available as market authorized product for the treatment of ADA-SCID (Strimvelis).

### Adenosine deaminase-deficient severe combined immunodeficiency

ADA-SCID is characterized by absence of all lymphoid cells, due to enzyme deficiency and toxic accumulation. Additionally, skeletal, nervous system, and liver abnormalities are often observed in patients [71]. Treatment options for ADA-SCID patients include enzyme replacement therapy, HSCT and gene therapy [72]. Enzyme replacement therapy allows stabilization of patients with metabolic detoxification, but comes with less efficient long-term immune reconstitution and has high financial costs [72].

Since 2016, Strimvelis, a gRV vector based product, has been available as market approved gene therapy for ADA-deficient patients without HLA-matched donor [14]. In contrast to other SCID patients treated with first generation gRV vectors, the rate of subsequent cancer development is much lower in ADA-SCID. After three decades, only 1 of at least 50 patients with ADA-SCID treated with a gRV vector based gene therapy, either on clinical trial [9,25,26,73,74^▪^,^75^,^76^] or with Strimvelis, has developed leukemia [77]. Detailed analysis showed integration in or near proto-oncogenes in more patients, but this did not lead to any leukemia [75]. The mystery of why ADA-SCID patients appear to have a much lower risk of insertional oncogenesis despite the use of gRV vectors remains unsolved [78]. In parallel, more than 50 ADA-SCID patients have been treated successfully with an LV vector [79^▪▪^]. Recent studies comparing gene therapy to mismatched donor HSCT for ADA-SCID showed a favorable survival of 100% versus 79.6%, respectively, with GvHD occurring in 24% of the HSCT patients [74^▪^,^76^].

### X-severe combined immunodeficiency

X-SCID is caused by pathogenic mutations in the X-linked gene IL2RG, that encodes the common gamma chain (γc) of the interleukin (IL)-2 receptor. Lack of γc-mediated signaling causes impaired development of T and NK cells, with presence of dysfunctional B cells. Initially, gRV vectors were used without conditioning, resulting in poor B cell reconstitution [7,12,13]. Six out of 20 treated patients developed acute T cell malignancies, due to integration near oncogenes, most notably LMO2 [12,34,36]. Further investigation showed that the therapeutic gene itself was not oncogenic [80]. This led to the development of safer SIN gRV viral vectors, with similar reconstitution as the previous gRV vectors [37]. Subsequent use of LV vector and low dose Busulfan conditioning led to improved T and B cell reconstitution [27,28]. Trials in the USA used an LV vector produced by St. Jude, with insulators flanking the codon optimized transgene. However, they showed clonal dominance in all 8 patients, which led to a voluntary hold of these trials (NCT01306019 and NCT01512888). Further safety analysis showed that the insulator used acts as a cryptic splice site resulting in truncated transcripts giving survival advantage to HMGA2 inserted clones [81]. Currently, two ongoing trials in the US and London are treating X-SCID patients with a different LV vector without any insulators in combinations with low dose busulfan conditioning (NCT03311503 and NCT03601286) with good B and T cell recovery [82,83].

### Artemis-deficient severe combined immunodeficiency

Artemis-deficient SCID is an autosomal recessive form of T-B-NK+SCID caused by pathogenic mutations in the *DCLRE1C* gene, encoding Artemis. This nuclease is critical for nonhomologous end joining of gene segments from Ig and TCR genes during recombination. Artemis is required to complete the last step of V(D)J rearrangement of the B and T cell receptor and in general for repair of DNA double strand breaks. Therefore, in addition to susceptibility to severe infections, Artemis patients are more prone to the toxic effects of ionizing radiation and alkylating chemotherapy. In particular, Artemis patients seem to suffer from the long-term toxic effects of conditioning chemotherapy, yet reduced conditioning leads to poor B and T cell reconstitution [84,85]. This led to the development of gene therapy with LV. An initial trial using low dose Busulfan showed good reconstitution and no insertional mutagenesis during follow up of 8–48 months, but longer follow up will be essential [86^▪▪^].

### Recombination activating genes 1/2 severe combined immunodeficiency

Recombination activating genes (*RAG*) 1 and 2 encode RAG1 and RAG2 proteins and are responsible for recombining the different V, (D) and J genes during B and T cell receptor rearrangement. Complete lack of functional RAG1 or RAG2 leads to T-B-NK+ SCID. As in other SCID genotypes, overall survival after HSCT with mismatched donors remains less favorable [17]. LV gene therapy with codon optimized RAG1 has been developed and after successful proof of concept results in RAG1 KO mice [87], a clinical trial has opened for RAG1-deficient SCID patients without an available HLA-matched donor (NCT04797260). The study is ongoing, with a favorable clinical course and immunological recovery in the first patients (unpublished results). For RAG2-SCID, a LV gene addition approach is being prepared for evaluation in a clinical trial (manuscript in preparation).

## GENE THERAPY FOR OTHER IEI THAN SEVERE COMBINED IMMUNODEFICIENCY

### Wiskott–Aldrich syndrome

Pathogenic mutations of the *WAS* gene cause an X-linked IEI resulting in defective actin polymerization and cytoskeletal remodeling affecting all hematopoietic lineages. This leads to complex immune dysregulation with eczema, thrombocytopenia and susceptibility to infections [88,89]. HSCT is the treatment of choice, with an overall survival of 89% [18]. The initial gene therapy trial for WAS used gRV vector and showed immunological reconstitution, but high incidence of insertional oncogenesis with seven out of nine patients developing leukemias, based on a similar mechanism as observed in the early X-SCID trials [32,33]. In subsequent studies with LV vectors no genotoxicity has been reported to date [90,91,92^▪^]. Immune improvement was favorable while platelet recovery was moderate, but high enough to prevent clinically significant bleeding.

### Neutrophil defects

Chronic granulomatous disease (CGD) is caused by mutations in the genes encoding subunits of the nicotinamide adenine dinucleotide phosphate (NADPH) complex resulting in dysfunctional production of reactive oxygen species and defective phagocytic function. The most common form of CGD is X-linked CGD caused by mutations in the *CYBB* gene encoding the gp91phox component. Mutations in the genes encoding other subunits of the NADPH complex are inherited in an autosomal recessive manner, the most common form of autosomal recessive CGD is caused by mutations in *NCF1*. Patients with CGD present with life threatening bacterial and fungal infections as well as uncontrolled inflammatory complications, in particular colitis [93]. Treatment of CGD is based on lifelong antibacterial and antifungal prophylaxis, at times combined with IFN-gamma treatment [94]. HSCT is curative with an overall survival of 86% for patients younger than 18 years of age and favorable outcomes in patients receiving HLA-matched stem cells [21]. Initial gene therapy trials for X-CGD with gRV vectors that were conducted thirty years ago led to correction of neutrophil function but were also complicated by vector-induced myelodysplastic syndrome [95]. More recent trials used LV vectors and showed long-term functional correction in the majority of patients without any signs of genotoxicity [96]. However, all four surviving pediatric patients treated in this trial showed initial neutrophil recovery followed by declining levels of corrected cells over the next months [96]. Another trial using LV vector showed a similar phenomenon and additional studies suggest that the inflammation affects quality and engraftment potential of the HSCs [97,98^▪▪^]. For autosomal recessive CGD (p47phox), preclinical studies in mice showed effective use of LV vector and a clinical trial has recently opened at the Great Ormond Street Hospital for Children with plans for an additional trial at the National Institutes of Health (NIH) (Table 1).

Leukocyte adhesion deficiency type 1 (LAD-1) is caused by recessive pathogenic mutations in the *ITGB2* gene, resulting in impaired expression of CD18, a crucial integrin molecule for neutrophil adhesion and migration [99]. Untreated patients develop early and severe bacterial infections with no purulent exudate formation as well as long term severe autoimmune complications [100]. HSCT leads to 75% overall survival [101]. Preclinical studies in animal models showed feasibility of gene therapy with a viral vector [102,103]. However, initial trials using gRV vector were unsuccessful and terminated early as no corrected cells were detected [104]. Currently, trials are being conducted using LV vectors in combination with busulfan conditioning, with interim reports showing successful reversal of the LAD-1 phenotype [105,106].

### Immune dysregulation, polyendocrinopathy enteropathy X-linked syndrome

Immune dysregulation, polyendocrinopathy enteropathy X-linked syndrome (IPEX) is caused by pathogenic mutations in the *FOXP3* gene. FOXP3 is a transcription factor required for the development of regulatory T cells (Tregs). Consequently, the syndrome is characterized by enteropathy and multiple autoimmune features. Treatment consists of supportive care, immune suppression and HSCT [107,108]. Using an LV vector to express FOXP3 in HSCs appeared to prevent T cells from differentiating into all required peripheral subsets [109]. However, mouse studies showed that using an LV-FOXP3 vector in CD4^+^ T cells resulted in functional Tregs and rescue of the scurfy phenotype [110,111]. Based on these preliminary studies, there is an open clinical trial for patients with IPEX syndrome at Lucile Packard Children's Hospital Stanford (Table 1).

### Hypomorphic recombination defects

When mutations in *DCLRE1C* or *RAG1* and *2* lead to residual activity, they can cause a broad immunological and clinical spectrum with different degrees of immunodeficiency and autoimmunity or inflammation [112–115]. HSCT is potentially curative in these patients, but more challenging than in individuals with SCID, because of increased risk for graft rejection due to residual T cell function. In addition, thymic abnormalities affecting mechanisms of immune tolerance have been described in patients with hypomorphic RAG mutations [116–118]. A recent retrospective study of HSCT in patients with hypomorphic RAG defects showed that poor pre-HSCT clinical status predicted an unfavorable HSCT outcome and slower naïve T-cell recovery, with especially low overall survival in the absence of an HLA matched donor [20]. Gene therapy might provide a strategy to overcome these hurdles. However, in patients with hypomorphic RAG deficiency uncorrected HSCs may retain the capacity to develop into defective peripheral T and B cells, a problem that is not encountered in uncorrected HSCs from SCID patients where there is a complete developmental block at an early progenitor stage [115].

## CHALLENGES AND FUTURE PERSPECTIVES

From 1995 to 2020 more than 200 patients with IEIs have been treated with gene therapy using viral gene addition, initially with gRV, later with self-inactivating (SIN) variants of gRV and LV [119]. These newer SIN vectors have been shown to be safer, with no hematological malignancies reported as yet in IEI patients. With improved protocols on HSC manipulation and preparative conditioning improving immune reconstitution, the number of successful gene therapy trials in IEI has grown significantly over the last decade. However, several challenges remain. Genes that are tightly regulated can be harmful when constitutively expressed with viral gene addition, as was shown for CD40L [57]. In such cases, gene editing with engineered nucleases would offer a promising alternative as it preserves endogenous gene regulation. Unfortunately, gene editing approaches tailor made for correction of a specific mutation are inefficient for IEI as patients generally show unique mutations and would each require a different gene therapy product. To date, several research groups have shown proof of principle for gene editing to correct IEI by adding an entire copy of the therapeutic gene to the same locus under the control of its endogenous promoter. In that case, the same approach would be applicable to all mutations. However, in contrast to delivery of the engineered nuclease, delivery of donor template is still challenging and has mostly been accomplished by use of nonintegrating viral vectors (AAV6) requiring additional safety studies [45,47,48,49^▪^,^51^–^53^,66^▪^].

For IEI that mainly affect T cells, modifying T cells is a good alternative. For IPEX, viral gene addition is being tested in a clinical trial [110,111] (Table 1). Gene editing of T cells instead of HSCs could be an attractive alternative as well. Gene editing of T cells reduces the risk of deleterious off-target mutagenesis as the cells are terminally differentiated and require less toxic conditioning for engraftment [120]. Proof of concept has been shown for CD40L deficiency [49^▪^], CTLA4 [52], IPEX [121] and XLP [53]. These studies also used donor template delivery by AAV6.

When gene therapy has been proven safe and effective in clinical trials, the next step is to make the therapy more widely available. Except for hospital exemption, currently the only option is to get market authorization and subsequent reimbursement. Gene therapy for ADA-SCID, Strimvelis (Orchard Therapeutics), was the first (and so far only) therapy to get this authorization in the EU. Remarkably, it was not reimbursed in all EU countries. Last year Orchard Therapeutics announced that they will discontinue gene therapy for WAS, X-CGD and ADA-SCID for commercial reasons [122^▪▪^,^123^]. It is becoming increasingly clear that the current for-profit model of market authorization and commercialization is not economically fit to achieve sustainable and affordable access to gene therapy for rare diseases such as IEI. To find solutions to these challenges the AGORA (Access to Gene Therapies for Rare Diseases) initiative was started comprising clinical academics, scientists and patient organizations [124] and a number of other national and international efforts are trying to obtain the same goal.

## CONCLUSION

A series of gene therapy trials using viral gene addition have been successful and gene therapy for ADA-SCID has been market authorized in Europe. Viral gene therapy approaches have become safer, more efficient and applicable to more IEI. Still, major obstacles for their further implementation and spread to many countries are health economics and complex regulations. Gene editing strategies to treat IEI are still at a preclinical level, but are promising alternatives for tightly regulated genes, gain-of-function mutations or dominant-negative mutations. Challenges for gene editing are minimizing on and off-site mutagenesis and optimizing correction efficiency. Current gene editing correction strategies applicable to multiple different mutations require a donor template containing the therapeutic gene. Delivering the donor template safe and efficiently to HSCs is still challenging.

## Acknowledgements

*None.*

### Financial support and sponsorship

*Our lab work is supported in part by a ZonMW E-RARE grant (40-419000-98-020) and EU H2020 grant RECOMB (755170-2) and has received funding from the European Union Horizon 2020 research and innovation program as well as from reNEW, the Novo Nordisk Foundation for Stem Cell Research (NNF21CC0073729).*

### Conflicts of interest

*L.O.B. receives grant support from NovoNordisk. FJTS receives grant support from Batavia Biosciences, NovoNordisk and Mustang Bio in accordance with the rules and guidelines by the Dutch Federations of Universities (NFU).*

## REFERENCES AND RECOMMENDED READING

Papers of particular interest, published within the annual period of review, have been highlighted as:

▪ of special interest
▪▪ of outstanding interest

## References

[1] Tangye SG Al-Herz W Bousfiha A. Human inborn errors of immunity: 2022 update on the classification from the International Union of Immunological Societies Expert Committee. J Clin Immunol 2022; 42:1473–1507. 35748970 10.1007/s10875-022-01289-3 PMC9244088

[2] Gatti RA Meuwissen HJ Allen HD. Immunological reconstitution of sex-linked lymphopenic immunological deficiency. Lancet 1968; 2:1366–1369. 4177932 10.1016/s0140-6736(68)92673-1

[3] De Koning J Van Bekkum DW Dicke KA. Transplantation of bone-marrow cells and fetal thymus in an infant with lymphopenic immunological deficiency. Lancet 1969; 1:1223–1227. 4182410 10.1016/s0140-6736(69)92112-6

[4] Hirschhorn R Yang DR Puck JM. Spontaneous in vivo reversion to normal of an inherited mutation in a patient with adenosine deaminase deficiency. Nat Genet 1996; 13:290–295. 8673127 10.1038/ng0796-290

[5] Stephan V Wahn V Le Deist F. Atypical X-linked severe combined immunodeficiency due to possible spontaneous reversion of the genetic defect in T cells. N Engl J Med 1996; 335:1563–1567. 8900089 10.1056/NEJM199611213352104

[6] Staal FJT Aiuti A Cavazzana M. Autologous stem-cell-based gene therapy for inherited disorders: state of the art and perspectives. Front Pediatr 2019; 7:443. 31737588 10.3389/fped.2019.00443 PMC6834641

[7] Cavazzana-Calvo M Hacein-Bey S de Saint Basile G. Gene therapy of human severe combined immunodeficiency (SCID)-X1 disease. Science 2000; 288:669–672. 10784449 10.1126/science.288.5466.669

[8] Aiuti A Slavin S Aker M. Correction of ADA-SCID by stem cell gene therapy combined with nonmyeloablative conditioning. Science 2002; 296:2410–2413. 12089448 10.1126/science.1070104

[9] Aiuti A Cattaneo F Galimberti S. Gene therapy for immunodeficiency due to adenosine deaminase deficiency. N Engl J Med 2009; 360:447–458. 19179314 10.1056/NEJMoa0805817

[10] Blaese RM Culver KW Chang L. Treatment of severe combined immunodeficiency disease (SCID) due to adenosine deaminase deficiency with CD34+ selected autologous peripheral blood cells transduced with a human ADA gene. Amendment to clinical research project, Project 90-C-195, January 10, 1992. Hum Gene Ther 1993; 4:521–527. 7691188 10.1089/hum.1993.4.4-521

[11] Kohn DB Weinberg KI Nolta JA. Engraftment of gene-modified umbilical cord blood cells in neonates with adenosine deaminase deficiency. Nat Med 1995; 1:1017–1023. 7489356 10.1038/nm1095-1017 PMC3013367

[12] Hacein-Bey-Abina S Le Deist F Carlier F. Sustained correction of X-linked severe combined immunodeficiency by ex vivo gene therapy. N Engl J Med 2002; 346:1185–1193. 11961146 10.1056/NEJMoa012616

[13] Gaspar HB Parsley KL Howe S. Gene therapy of X-linked severe combined immunodeficiency by use of a pseudotyped gammaretroviral vector. Lancet 2004; 364:2181–2187. 15610804 10.1016/S0140-6736(04)17590-9

[14] Aiuti A Roncarolo MG Naldini L. Gene therapy for ADA-SCID, the first marketing approval of an ex vivo gene therapy in Europe: paving the road for the next generation of advanced therapy medicinal products. EMBO Mol Med 2017; 9:737–740. 28396566 10.15252/emmm.201707573 PMC5452047

[15] Pai SY Logan BR Griffith LM. Transplantation outcomes for severe combined immunodeficiency, 2000–2009. N Engl J Med 2014; 371:434–446. 25075835 10.1056/NEJMoa1401177 PMC4183064

[16] Gennery AR Lankester A Marrow T. Inborn Errors Working Party of the European Society for B. Long term outcome and immune function after hematopoietic stem cell transplantation for primary immunodeficiency. Front Pediatr 2019; 7:381. 31616648 10.3389/fped.2019.00381 PMC6768963

[17] Lankester AC Neven B Mahlaoui N. Hematopoietic cell transplantation in severe combined immunodeficiency: the SCETIDE 2006–2014 European cohort. J Allergy Clin Immunol 2022; 149:1744–1754. e8. 34718043 10.1016/j.jaci.2021.10.017

[18] Albert MH Slatter MA Gennery AR. Hematopoietic stem cell transplantation for Wiskott–Aldrich syndrome: an EBMT Inborn Errors Working Party analysis. Blood 2022; 139:2066–2079. 35100336 10.1182/blood.2021014687

[19] Dedieu C Albert MH Mahlaoui N. Outcome of chronic granulomatous disease – conventional treatment vs stem cell transplantation. Pediatr Allergy Immunol 2021; 32:576–585. 33118209 10.1111/pai.13402

[20] Schuetz C Gerke J Ege M. Hypomorphic RAG deficiency: impact of disease burden on survival and thymic recovery argues for early diagnosis and HSCT. Blood 2023; 141:713–724. 36279417 10.1182/blood.2022017667 PMC10082356

[21] Chiesa R Wang J Blok HJ. Hematopoietic cell transplantation in chronic granulomatous disease: a study of 712 children and adults. Blood 2020; 136:1201–1211. 32614953 10.1182/blood.2020005590

[22] Tajer P Pike-Overzet K Arias S. Ex vivo expansion of hematopoietic stem cells for therapeutic purposes: lessons from development and the niche. Cells 2019; 8:10.3390/cells8020169 PMC6407064 30781676

[23] Lidonnici MR Aprile A Frittoli MC. Plerixafor and G-CSF combination mobilizes hematopoietic stem and progenitors cells with a distinct transcriptional profile and a reduced in vivo homing capacity compared to plerixafor alone. Haematologica 2017; 102:e120–e124. 28034992 10.3324/haematol.2016.154740 PMC5395121

[24] Anasetti C Logan BR Lee SJ. Peripheral-blood stem cells versus bone marrow from unrelated donors. N Engl J Med 2012; 367:1487–1496. 23075175 10.1056/NEJMoa1203517 PMC3816375

[25] Gaspar HB Cooray S Gilmour KC. Hematopoietic stem cell gene therapy for adenosine deaminase-deficient severe combined immunodeficiency leads to long-term immunological recovery and metabolic correction. Sci Transl Med 2011; 3: 97ra80. 10.1126/scitranslmed.3002716 21865538

[26] Candotti F Shaw KL Muul L. Gene therapy for adenosine deaminase-deficient severe combined immune deficiency: clinical comparison of retroviral vectors and treatment plans. Blood 2012; 120:3635–3646. 22968453 10.1182/blood-2012-02-400937 PMC3488882

[27] De Ravin SS Wu X Moir S. Lentiviral hematopoietic stem cell gene therapy for X-linked severe combined immunodeficiency. Sci Transl Med 2016; 8: 335ra57. 10.1126/scitranslmed.aad8856 PMC5557273 27099176

[28] Mamcarz E Zhou S Lockey T. Lentiviral gene therapy combined with low-dose busulfan in infants with SCID-X1. N Engl J Med 2019; 380:1525–1534. 30995372 10.1056/NEJMoa1815408 PMC6636624

[29] Bernardo ME Aiuti A. The role of conditioning in hematopoietic stem-cell gene therapy. Hum Gene Ther 2016; 27:741–748. 27530055 10.1089/hum.2016.103

[30] Milone MC O’Doherty U. Clinical use of lentiviral vectors. Leukemia 2018; 32:1529–1541. 29654266 10.1038/s41375-018-0106-0 PMC6035154

[31] Garcia-Perez L van Eggermond M Maietta E. A novel branched DNA-based flowcytometric method for single-cell characterization of gene therapy products and expression of therapeutic genes. Front Immunol 2020; 11:607991. 33584681 10.3389/fimmu.2020.607991 PMC7876092

[32] Boztug K Schmidt M Schwarzer A. Stem-cell gene therapy for the Wiskott–Aldrich syndrome. N Engl J Med 2010; 363:1918–1927. 21067383 10.1056/NEJMoa1003548 PMC3064520

[33] Braun CJ Boztug K Paruzynski A. Gene therapy for Wiskott–Aldrich syndrome—long-term efficacy and genotoxicity. Sci Transl Med 2014; 6: 227ra33. 10.1126/scitranslmed.3007280 24622513

[34] Hacein-Bey-Abina S Von Kalle C Schmidt M. LMO2-associated clonal T cell proliferation in two patients after gene therapy for SCID-X1. Science 2003; 302:415–419. 14564000 10.1126/science.1088547

[35] Hacein-Bey-Abina S Garrigue A Wang GP. Insertional oncogenesis in 4 patients after retrovirus-mediated gene therapy of SCID-X1. J Clin Invest 2008; 118:3132–3142. 18688285 10.1172/JCI35700 PMC2496963

[36] Howe SJ Mansour MR Schwarzwaelder K. Insertional mutagenesis combined with acquired somatic mutations causes leukemogenesis following gene therapy of SCID-X1 patients. J Clin Invest 2008; 118:3143–3150. 18688286 10.1172/JCI35798 PMC2496964

[37] Hacein-Bey-Abina S Pai SY Gaspar HB. A modified gamma-retrovirus vector for X-linked severe combined immunodeficiency. N Engl J Med 2014; 371:1407–1417. 25295500 10.1056/NEJMoa1404588 PMC4274995

[38] Arlabosse T Booth C Candotti F. Gene therapy for inborn errors of immunity. J Allergy Clin Immunol Pract 2023; 11:1592–1601. 37084938 10.1016/j.jaip.2023.04.001

[39] Schwarzer A Talbot SR Selich A. Predicting genotoxicity of viral vectors for stem cell gene therapy using gene expression-based machine learning. Mol Ther 2021; 29:3383–3397. 34174440 10.1016/j.ymthe.2021.06.017 PMC8636173

[40] Ferrari S Vavassori V Canarutto D. Gene editing of hematopoietic stem cells: hopes and hurdles toward clinical translation. Front Genome Ed 2021; 3:618378. 34713250 10.3389/fgeed.2021.618378 PMC8525369

[41] De Ravin SS Brault J Meis RJ. Enhanced homology-directed repair for highly efficient gene editing in hematopoietic stem/progenitor cells. Blood 2021; 137:2598–2608. 33623984 10.1182/blood.2020008503 PMC8120141

[42] De Ravin SS Li L Wu X. CRISPR-Cas9 gene repair of hematopoietic stem cells from patients with X-linked chronic granulomatous disease. Sci Transl Med 2017; 9:10.1126/scitranslmed.aah3480 28077679

[43] Gardner CL Pavel-Dinu M Dobbs K. Gene editing rescues in vitro T cell development of RAG2-deficient induced pluripotent stem cells in an artificial thymic organoid system. J Clin Immunol 2021; 41:852–862. 33650026 10.1007/s10875-021-00989-6 PMC8254788

[44] Mara Pavel-Dinu CLG Yusuke Nakauchi Tomoki Kawai. Genetically corrected RAG2-SCID human hematopoietic stem cells restore V(D)J-recombinase and rescue lymphoid deficiency. BioRxiv 2022. 10.1182/bloodadvances.2023011766 PMC11006817 38096800

[45] Pavel-Dinu M Wiebking V Dejene BT. Gene correction for SCID-X1 in long-term hematopoietic stem cells. Nat Commun 2019; 10:1634. 30967552 10.1038/s41467-019-09614-y PMC6456568

[46] Schiroli G Ferrari S Conway A. Preclinical modeling highlights the therapeutic potential of hematopoietic stem cell gene editing for correction of SCID-X1. Sci Transl Med 2017; 9:10.1126/scitranslmed.aan0820 29021165

[47] Brault J Liu T Liu S. CRISPR-Cas9-AAV versus lentivector transduction for genome modification of X-linked severe combined immunodeficiency hematopoietic stem cells. Front Immunol 2022; 13:1067417. 36685559 10.3389/fimmu.2022.1067417 PMC9846165

[48] Sweeney CL Pavel-Dinu M Choi U. Correction of X-CGD patient HSPCs by targeted CYBB cDNA insertion using CRISPR/Cas9 with 53BP1 inhibition for enhanced homology-directed repair. Gene Ther 2021; 28:373–390. 33712802 10.1038/s41434-021-00251-z PMC8232036

[49▪] Vavassori V Mercuri E Marcovecchio GE. Modeling, optimization, and comparable efficacy of T cell and hematopoietic stem cell gene editing for treating hyper-IgM syndrome. EMBO Mol Med 2021; 13:e13545. 33475257 10.15252/emmm.202013545 PMC7933961

[50] Hubbard N Hagin D Sommer K. Targeted gene editing restores regulated CD40L function in X-linked hyper-IgM syndrome. Blood 2016; 127:2513–2522. 26903548 10.1182/blood-2015-11-683235

[51] Kuo CY Long JD Campo-Fernandez B. Site-specific gene editing of human hematopoietic stem cells for X-linked hyper-IgM syndrome. Cell Rep 2018; 23:2606–2616. 29847792 10.1016/j.celrep.2018.04.103 PMC6181643

[52] Fox TA Houghton BC Petersone L. Therapeutic gene editing of T cells to correct CTLA-4 insufficiency. Sci Transl Med 2022; 14:eabn5811. 36288278 10.1126/scitranslmed.abn5811 PMC7617859

[53] Houghton BC Panchal N Haas SA. Genome editing with TALEN, CRISPR-Cas9 and CRISPR-Cas12a in combination with AAV6 homology donor restores T cell function for XLP. Front Genome Ed 2022; 4:828489. 35677600 10.3389/fgeed.2022.828489 PMC9168036

[54] Castiello NS, Draghici E, Ferrari S, *et al*. Targeted genome editing of hematopoietic stem cells for treating recombination activating gene 1 (RAG1) immunodeficiency. ASGCT; 2021;1–427.

[55] Rhiel JK, Jullierat A, Andrieux G, editors. Preclinical development of a TALEN®-based genome editing therapy for RAG1 deficiency. ESGCT; 2021.

[56] Rai R Romito M Rivers E. Targeted gene correction of human hematopoietic stem cells for the treatment of Wiskott–Aldrich Syndrome. Nat Commun 2020; 11:4034. 32788576 10.1038/s41467-020-17626-2 PMC7423939

[57] Brown MP Topham DJ Sangster MY. Thymic lymphoproliferative disease after successful correction of CD40 ligand deficiency by gene transfer in mice. Nat Med 1998; 4:1253–1260. 9809548 10.1038/3233

[58] Sacco MG Ungari M Cato EM. Lymphoid abnormalities in CD40 ligand transgenic mice suggest the need for tight regulation in gene therapy approaches to hyper immunoglobulin M (IgM) syndrome. Cancer Gene Ther 2000; 7:1299–1306. 11059686 10.1038/sj.cgt.7700232

[59] De Ravin SS Reik A Liu PQ. Targeted gene addition in human CD34(+) hematopoietic cells for correction of X-linked chronic granulomatous disease. Nat Biotechnol 2016; 34:424–429. 26950749 10.1038/nbt.3513 PMC4824656

[60] Ferrari G Thrasher AJ Aiuti A. Gene therapy using haematopoietic stem and progenitor cells. Nat Rev Genet 2021; 22:216–234. 33303992 10.1038/s41576-020-00298-5

[61] Ott de Bruin LM Volpi S Musunuru K. Novel genome-editing tools to model and correct primary immunodeficiencies. Front Immunol 2015; 6:250. 26052330 10.3389/fimmu.2015.00250 PMC4440404

[62] Wang JY Doudna JA. CRISPR technology: a decade of genome editing is only the beginning. Science 2023; 379:eadd8643. 36656942 10.1126/science.add8643

[63] Bao XR Pan Y Lee CM. Tools for experimental and computational analyses of off-target editing by programmable nucleases. Nat Protoc 2021; 16:10–26. 33288953 10.1038/s41596-020-00431-y PMC8049448

[64] Turchiano G Andrieux G Klermund J. Quantitative evaluation of chromosomal rearrangements in gene-edited human stem cells by CAST-Seq. Cell Stem Cell 2021; 28:1136–1147. e5. 33626327 10.1016/j.stem.2021.02.002

[65] Wang D Zhang F Gao G. CRISPR-based therapeutic genome editing: strategies and in vivo delivery by AAV vectors. Cell 2020; 181:136–150. 32243786 10.1016/j.cell.2020.03.023 PMC7236621

[66▪] Ferrari S Jacob A Cesana D. Choice of template delivery mitigates the genotoxic risk and adverse impact of editing in human hematopoietic stem cells. Cell Stem Cell 2022; 29:1428–1444. e9. 36206730 10.1016/j.stem.2022.09.001 PMC9550218

[67] Newby GA Liu DR. In vivo somatic cell base editing and prime editing. Mol Ther 2021; 29:3107–3124. 34509669 10.1016/j.ymthe.2021.09.002 PMC8571176

[68] Anzalone AV Koblan LW Liu DR. Genome editing with CRISPR-Cas nucleases, base editors, transposases and prime editors. Nat Biotechnol 2020; 38:824–844. 32572269 10.1038/s41587-020-0561-9

[69] Eberherr AC Maaske A Wolf C. Rescue of STAT3 function in hyper-IgE syndrome using adenine base editing. CRISPR J 2021; 4:178–190. 33876960 10.1089/crispr.2020.0111

[70] Currier R Puck JM. SCID newborn screening: what we’ve learned. J Allergy Clin Immunol 2021; 147:417–426. 33551023 10.1016/j.jaci.2020.10.020 PMC7874439

[71] Bradford KL Moretti FA Carbonaro-Sarracino DA. Adenosine deaminase (ADA)-deficient severe combined immune deficiency (SCID): molecular pathogenesis and clinical manifestations. J Clin Immunol 2017; 37:626–637. 28842866 10.1007/s10875-017-0433-3

[72] Grunebaum E Booth C Cuvelier GDE. Updated management guidelines for adenosine deaminase deficiency. J Allergy Clin Immunol Pract 2023; 11:1665–1675. 36736952 10.1016/j.jaip.2023.01.032

[73] Shaw KL Garabedian E Mishra S. Clinical efficacy of gene-modified stem cells in adenosine deaminase-deficient immunodeficiency. J Clin Invest 2017; 127:1689–1699. 28346229 10.1172/JCI90367 PMC5409097

[74▪] Cuvelier GDE Logan BR Prockop SE. Outcomes following treatment for ADA-deficient severe combined immunodeficiency: a report from the PIDTC. Blood 2022; 140:685–705. 35671392 10.1182/blood.2022016196 PMC9389638

[75] Reinhardt B Habib O Shaw KL. Long-term outcomes after gene therapy for adenosine deaminase severe combined immune deficiency. Blood 2021; 138:1304–1316. 33974038 10.1182/blood.2020010260 PMC8525336

[76] Kuo CY Garabedian E Puck J. Adenosine deaminase (ADA)-deficient severe combined immune deficiency (SCID) in the US Immunodeficiency Network (USIDNet) Registry. J Clin Immunol 2020; 40:1124–1131. 32880085 10.1007/s10875-020-00857-9 PMC8216639

[77] Orchard Therapeutics’ gene therapy Strimvelis linked to a leukemia case [updated Nov 2, 2020[. Available at: https://www.fiercepharma.com/pharma/orchard-s-rare-disease-gene-therapy-strimvelis-linked-to-a-leukemia-case.

[78] Pai SY. Built to last: gene therapy for ADA SCID. Blood 2021; 138:1287–1288. 34647983 10.1182/blood.2021012300 PMC8525332

[79▪▪] Kohn DB Booth C Shaw KL. Autologous ex vivo lentiviral gene therapy for adenosine deaminase deficiency. N Engl J Med 2021; 384:2002–2013. 33974366 10.1056/NEJMoa2027675 PMC8240285

[80] Pike-Overzet K de Ridder D Weerkamp F. Gene therapy: is IL2RG oncogenic in T-cell development? Nature 2006; 443:E5 discussion E6–7. 10.1038/nature05218 16988660

[81] De Ravin SS Liu S Sweeney CL. Lentivector cryptic splicing mediates increase in CD34+ clones expressing truncated HMGA2 in human X-linked severe combined immunodeficiency. Nat Commun 2022; 13:3710. 35764638 10.1038/s41467-022-31344-x PMC9240040

[82] Booth C Kohn DB Armant M. Lentiviral gene therapy with low dose conditioning for X-linked SCID results in complete immune reconstitution and no evidence of clonal expansion. Blood 2022; 140:7770–7771.

[83] Sung-Yun Pai CB Kohn DB Armant MA. Universal survival and superior immune reconstitution after lentiviral gene therapy with low dose conditioning for X-linked SCID (SCID-X1). Mol Ther 2023; 31:1–8.36528029

[84] Schuetz C Neven B Dvorak CC. SCID patients with ARTEMIS vs RAG deficiencies following HCT: increased risk of late toxicity in ARTEMIS-deficient SCID. Blood 2014; 123:281–289. 24144642 10.1182/blood-2013-01-476432 PMC3953035

[85] Haddad E Logan BR Griffith LM. SCID genotype and 6-month posttransplant CD4 count predict survival and immune recovery. Blood 2018; 132:1737–1749. 30154114 10.1182/blood-2018-03-840702 PMC6202916

[86▪▪] Cowan MJ Yu J Facchino J. Lentiviral gene therapy for Artemis-deficient SCID. N Engl J Med 2022; 387:2344–2355. 36546626 10.1056/NEJMoa2206575 PMC9884487

[87] Garcia-Perez L van Eggermond M van Roon L. Successful preclinical development of gene therapy for recombinase-activating gene-1-deficient SCID. Mol Ther Methods Clin Dev 2020; 17:666–682. 32322605 10.1016/j.omtm.2020.03.016 PMC7163047

[88] Derry JM Ochs HD Francke U. Isolation of a novel gene mutated in Wiskott–Aldrich syndrome. Cell 1994; 78:635–644. 8069912 10.1016/0092-8674(94)90528-2

[89] Candotti F. Clinical manifestations and pathophysiological mechanisms of the Wiskott–Aldrich syndrome. J Clin Immunol 2018; 38:13–27. 29086100 10.1007/s10875-017-0453-z

[90] Hacein-Bey Abina S Gaspar HB Blondeau J. Outcomes following gene therapy in patients with severe Wiskott–Aldrich syndrome. JAMA 2015; 313:1550–1563. 25898053 10.1001/jama.2015.3253 PMC4942841

[91] Ferrua F Cicalese MP Galimberti S. Lentiviral haemopoietic stem/progenitor cell gene therapy for treatment of Wiskott–Aldrich syndrome: interim results of a nonrandomised, open-label, phase 1/2 clinical study. Lancet Haematol 2019; 6:e239–e253. 30981783 10.1016/S2352-3026(19)30021-3 PMC6494976

[92▪] Magnani A Semeraro M Adam F. Long-term safety and efficacy of lentiviral hematopoietic stem/progenitor cell gene therapy for Wiskott-Aldrich syndrome. Nat Med 2022; 28:71–80. 35075289 10.1038/s41591-021-01641-x PMC8799465

[93] Holland SM. Chronic granulomatous disease. Hematol Oncol Clin North Am 2013; 27:89–99. viii. 23351990 10.1016/j.hoc.2012.11.002 PMC3558921

[94] International Chronic Granulomatous Disease Cooperative Study Group. A controlled trial of interferon gamma to prevent infection in chronic granulomatous disease. N Engl J Med 1991; 324:509–516. 1846940 10.1056/NEJM199102213240801

[95] Ott MG Schmidt M Schwarzwaelder K. Correction of X-linked chronic granulomatous disease by gene therapy, augmented by insertional activation of MDS1-EVI1, PRDM16 or SETBP1. Nat Med 2006; 12:401–409. 16582916 10.1038/nm1393

[96] Kohn DB Booth C Kang EM. Lentiviral gene therapy for X-linked chronic granulomatous disease. Nat Med 2020; 26:200–206. 31988463 10.1038/s41591-019-0735-5 PMC7115833

[97] Weisser M Demel UM Stein S. Hyperinflammation in patients with chronic granulomatous disease leads to impairment of hematopoietic stem cell functions. J Allergy Clin Immunol 2016; 138:219–228. e9. 26853280 10.1016/j.jaci.2015.11.028

[98▪▪] Sobrino S Magnani A Semeraro M. Severe hematopoietic stem cell inflammation compromises chronic granulomatous disease gene therapy. Cell Rep Med 2023; 4:100919. 36706754 10.1016/j.xcrm.2023.100919 PMC9975109

[99] Kishimoto TK Hollander N Roberts TM. Heterogeneous mutations in the beta subunit common to the LFA-1, Mac-1, and p150,95 glycoproteins cause leukocyte adhesion deficiency. Cell 1987; 50:193–202. 3594570 10.1016/0092-8674(87)90215-7

[100] De Rose DU Giliani S Notarangelo LD. Long term outcome of eight patients with type 1 leukocyte adhesion deficiency (LAD-1): not only infections, but high risk of autoimmune complications. Clin Immunol 2018; 191:75–80. 29548898 10.1016/j.clim.2018.03.005

[101] Qasim W Cavazzana-Calvo M Davies EG. Allogeneic hematopoietic stem-cell transplantation for leukocyte adhesion deficiency. Pediatrics 2009; 123:836–840. 19255011 10.1542/peds.2008-1191 PMC3380632

[102] Mesa-Nunez C Damian C Fernandez-Garcia M. Preclinical safety and efficacy of lentiviral-mediated gene therapy for leukocyte adhesion deficiency type I. Mol Ther Methods Clin Dev 2022; 26:459–470. 36092365 10.1016/j.omtm.2022.07.015 PMC9418989

[103] Hunter MJ Tuschong LM Fowler CJ. Gene therapy of canine leukocyte adhesion deficiency using lentiviral vectors with human CD11b and CD18 promoters driving canine CD18 expression. Mol Ther 2011; 19:113–121. 20859258 10.1038/mt.2010.203 PMC3017439

[104] Bauer TR Jr Hickstein DD. Gene therapy for leukocyte adhesion deficiency. Curr Opin Mol Ther 2000; 2:383–388. 11249768

[105] Kohn DB Sevilla J Rao G. Interim results from an ongoing phase 1/2 study of lentiviral-mediated ex-vivo gene therapy for pediatric patients with severe leukocyte adhesion deficiency-I (LAD-I). Mol Ther 2022; 30:550.34478871

[106] Booth C Sevilla J Rao GR. Interim results from an ongoing phase 1/2 study of lentiviral-mediated ex-vivo gene therapy for pediatric patients with severe leukocyte adhesion deficiency-I (LAD-I). Blood 2022; 140:7774–7775.

[107] Bacchetta R Barzaghi F Roncarolo MG. From IPEX syndrome to FOXP3 mutation: a lesson on immune dysregulation. Ann N Y Acad Sci 2018; 1417:5–22. 26918796 10.1111/nyas.13011

[108] Kucuk ZY Bleesing JJ Marsh R. A challenging undertaking: stem cell transplantation for immune dysregulation, polyendocrinopathy, enteropathy, X-linked (IPEX) syndrome. J Allergy Clin Immunol 2016; 137:953–955. e4. 26559324 10.1016/j.jaci.2015.09.030

[109] Santoni de Sio FR Passerini L Valente MM. Ectopic FOXP3 expression preserves primitive features of human hematopoietic stem cells while impairing functional T cell differentiation. Sci Rep 2017; 7:15820. 29150659 10.1038/s41598-017-15689-8 PMC5693945

[110] Passerini L Rossi Mel E Sartirana C. CD4(+) T cells from IPEX patients convert into functional and stable regulatory T cells by FOXP3 gene transfer. Sci Transl Med 2013; 5: 215ra174. 10.1126/scitranslmed.3007320 24337481

[111] Sato Y Passerini L Piening BD. Human-engineered Treg-like cells suppress FOXP3-deficient T cells but preserve adaptive immune responses in vivo. Clin Transl Immunol 2020; 9:e1214. 10.1002/cti2.1214 PMC7688376 33304583

[112] Notarangelo LD Kim MS Walter JE Lee YN. Human RAG mutations: biochemistry and clinical implications. Nat Rev Immunol 2016; 16:234–246. 26996199 10.1038/nri.2016.28 PMC5757527

[113] Felgentreff K Lee YN Frugoni F. Functional analysis of naturally occurring DCLRE1C mutations and correlation with the clinical phenotype of ARTEMIS deficiency. J Allergy Clin Immunol 2015; 136:140–150. e7. 25917813 10.1016/j.jaci.2015.03.005 PMC4494888

[114] Volk T Pannicke U Reisli I. DCLRE1C (ARTEMIS) mutations causing phenotypes ranging from atypical severe combined immunodeficiency to mere antibody deficiency. Hum Mol Genet 2015; 24:7361–7372. 26476407 10.1093/hmg/ddv437 PMC4664172

[115] Ott de Bruin LM Bosticardo M Barbieri A. Hypomorphic Rag1 mutations alter the preimmune repertoire at early stages of lymphoid development. Blood 2018; 132:281–292. 29743177 10.1182/blood-2017-12-820985 PMC6053949

[116] Poliani PL Facchetti F Ravanini M. Early defects in human T-cell development severely affect distribution and maturation of thymic stromal cells: possible implications for the pathophysiology of Omenn syndrome. Blood 2009; 114:105–108. 19414857 10.1182/blood-2009-03-211029 PMC2710940

[117] De Ravin SS Cowen EW Zarember KA. Hypomorphic Rag mutations can cause destructive midline granulomatous disease. Blood 2010; 116:1263–1271. 20489056 10.1182/blood-2010-02-267583 PMC2938237

[118] Cavadini P Vermi W Facchetti F. AIRE deficiency in thymus of 2 patients with Omenn syndrome. J Clin Invest 2005; 115:728–732. 15696198 10.1172/JCI23087 PMC546458

[119] Tucci F Galimberti S Naldini L. A systematic review and meta-analysis of gene therapy with hematopoietic stem and progenitor cells for monogenic disorders. Nat Commun 2022; 13:1315. 35288539 10.1038/s41467-022-28762-2 PMC8921234

[120] Fox TA Houghton BC Booth C. Gene edited T cell therapies for inborn errors of immunity. Front Genome Ed 2022; 4:899294. 35783679 10.3389/fgeed.2022.899294 PMC9244397

[121] Honaker Y Hubbard N Xiang Y. Gene editing to induce FOXP3 expression in human CD4(+) T cells leads to a stable regulatory phenotype and function. Sci Transl Med 2020; 12:10.1126/scitranslmed.aay6422 32493794

[122▪▪] Aiuti A Pasinelli F Naldini L. Ensuring a future for gene therapy for rare diseases. Nat Med 2022; 28:1985–1988. 35970921 10.1038/s41591-022-01934-9

[123] Orchard Therapeutics Extends Runway into 2024, Focusing HSC Gene Therapy Platform Exclusively on Severe Neurometabolic Diseases and Research Platform 202.2 Available at: https://ir.orchard-tx.com/node/8771/pdf.

[124] Fox T Bueren J Candotti F. Access to gene therapy for rare diseases when commercialization is not fit for purpose. Nat Med 2023; 29:518–519. 36782029 10.1038/s41591-023-02208-8

[125] Pike-Overzet K Rodijk M Ng YY. Correction of murine Rag1 deficiency by self-inactivating lentiviral vector-mediated gene transfer. Leukemia 2011; 25:1471–1483. 21617701 10.1038/leu.2011.106

[126] van Til NP de Boer H Mashamba N. Correction of murine Rag2 severe combined immunodeficiency by lentiviral gene therapy using a codon-optimized RAG2 therapeutic transgene. Mol Ther 2012; 20:1968–1980. 22692499 10.1038/mt.2012.110 PMC3464632

[127] Capo V Castiello MC Fontana E. Efficacy of lentivirus-mediated gene therapy in an Omenn syndrome recombination-activating gene 2 mouse model is not hindered by inflammation and immune dysregulation. J Allergy Clin Immunol 2018; 142:928–941. e8. 29241731 10.1016/j.jaci.2017.11.015 PMC6081264

[128] Ghosh S Carmo M Calero-Garcia M. T-cell gene therapy for perforin deficiency corrects cytotoxicity defects and prevents hemophagocytic lymphohistiocytosis manifestations. J Allergy Clin Immunol 2018; 142:904–913. e3. 29355678 10.1016/j.jaci.2017.11.050 PMC6127027

[129] Carmo M Risma KA Arumugam P. Perforin gene transfer into hematopoietic stem cells improves immune dysregulation in murine models of perforin deficiency. Mol Ther 2015; 23:737–745. 25523759 10.1038/mt.2014.242 PMC4395774

[130] Dettmer V Bloom K Gross M. Retroviral UNC13D gene transfer restores cytotoxic activity of T cells derived from familial hemophagocytic lymphohistiocytosis type 3 patients in vitro. Hum Gene Ther 2019; 30:975–984. 31032638 10.1089/hum.2019.025

[131] Soheili T Riviere J Ricciardelli I. Gene-corrected human Munc13-4-deficient CD8+ T cells can efficiently restrict EBV-driven lymphoproliferation in immunodeficient mice. Blood 2016; 128:2859–2862. 27799161 10.1182/blood-2016-07-729871

[132] Soheili T Durand A Sepulveda FE. Gene transfer into hematopoietic stem cells reduces HLH manifestations in a murine model of Munc13-4 deficiency. Blood Adv 2017; 1:2781–2789. 29296930 10.1182/bloodadvances.2017012088 PMC5745141

[133] Takushi SE Paik NY Fedanov A. Lentiviral gene therapy for familial hemophagocytic lymphohistiocytosis type 3, caused by UNC13D genetic defects. Hum Gene Ther 2020; 31:626–638. 32253931 10.1089/hum.2019.329 PMC7310202

[134] Topal J Panchal N Barroeta A. Lentiviral gene transfer corrects immune abnormalities in XIAP deficiency. J Clin Immunol 2023; 43:440–451. 36329240 10.1007/s10875-022-01389-0 PMC9892131

[135] Rivat C Booth C Alonso-Ferrero M. SAP gene transfer restores cellular and humoral immune function in a murine model of X-linked lymphoproliferative disease. Blood 2013; 121:1073–1076. 23223356 10.1182/blood-2012-07-445858 PMC3779401

[136] Panchal N Houghton B Diez B. Transfer of gene-corrected T cells corrects humoral and cytotoxic defects in patients with X-linked lymphoproliferative disease. J Allergy Clin Immunol 2018; 142:235–245. e6. 29705247 10.1016/j.jaci.2018.02.053 PMC6034012

[137] Ng YY Baert MR Pike-Overzet K. Correction of B-cell development in Btk-deficient mice using lentiviral vectors with codon-optimized human BTK. Leukemia 2010; 24:1617–1630. 20574453 10.1038/leu.2010.140

[138] Seymour BJ Singh S Certo HM. Effective, safe, and sustained correction of murine XLA using a UCOE-BTK promoter-based lentiviral vector. Mol Ther Methods Clin Dev 2021; 20:635–651. 33718514 10.1016/j.omtm.2021.01.007 PMC7907679

[139] Lagresle-Peyrou C Six EM Picard C. Human adenylate kinase 2 deficiency causes a profound hematopoietic defect associated with sensorineural deafness. Nat Genet 2009; 41:106–111. 19043416 10.1038/ng.278 PMC2612090

